# Wuji Wan Formula Ameliorates Diarrhea and Disordered Colonic Motility in Post-inflammation Irritable Bowel Syndrome Rats by Modulating the Gut Microbiota

**DOI:** 10.3389/fmicb.2017.02307

**Published:** 2017-11-23

**Authors:** Ying Chen, Shuiming Xiao, Zipeng Gong, Xiaoxin Zhu, Qing Yang, Yujie Li, Shuangrong Gao, Yu Dong, Zhe Shi, Yajie Wang, Xiaogang Weng, Qi Li, Weiyan Cai, Weijie Qiang

**Affiliations:** ^1^Institute of Chinese Materia Medica, China Academy of Chinese Medical Sciences, Beijing, China; ^2^Provincial Key Laboratory of Pharmaceutics in Guizhou Province, School of Pharmacy, Guiyang Medical University, Guiyang, China; ^3^Guang’an Men Hospital, China Academy of Chinese Medical Sciences, Beijing, China

**Keywords:** Wuji Wan, post-inflammation irritable bowel syndrome, gut microbiota, mucin, tight junctions

## Abstract

Emerging evidence suggests that gut microbiota contribute to the treatment of post-inflammatory irritable bowel syndrome (PI-IBS). Our previous studies have demonstrated that a Chinese formula, Wuji Wan, has the ability to mitigate abdominal pain and diarrhea in PI-IBS rats. However, little is known about the underlying mechanism and whether the gut microbiota mediate the effect of Wuji Wan on PI-IBS. Thus, the aim of this study was to determine whether Wuji Wan mitigated PI-IBS by modifying the gut microbiota. PI-IBS was induced in Sprague-Dawley rats by enema using 4% acetic acid and restraint stress. Rats were fed water, Wuji Wan extract (630 mg/kg) or pinaverium bromide (13.5 mg/kg). Our data showed that Wuji Wan effectively ameliorated abdominal pain, colonic motility abnormality and visceral hypersensitivity. Analysis of the fecal microbiota showed that Wuji Wan could reverse the reduction in richness of the gut microbiota and significantly increase the relative abundances of *Akkermansia, Bacteroides*, and *Parasutterella*; however, *Lactobacillus* and *Prevotella* were markedly decreased in the PI-IBS rats. Moreover, Wuji Wan promoted goblet cell proliferation in the colonic mucosa by increasing the release of mucin, up-regulating the distribution of tight junction proteins Occludin and ZO-1 and down-regulating the expression of MLCK in colonic epithelial cells. These findings suggest that Wuji Wan may remit IBS by modulating the gut microbiota and stabilizing the gut mucosal barrier, indicating that the use of a classical formula of Traditional Chinese Medicine (TCM) that exhibits a prebiotic effect may be a promising strategy for PI-IBS treatment.

## Introduction

Irritable bowel syndrome (IBS) comprises multiple functional bowel disorders, including recurrent abdominal pain associated with discomfort, diarrhea or constipation, and the prevalence of IBS is between 5 and 20% of the population worldwide and between 5 and 10% of Chinese adults (Rivkin, 2003). IBS is a common complication of gastrointestinal disease without substantial organic lesions. Although the exact etiology of IBS remains poorly understood, evidence indicates that several factors may account for its pathogenesis, including inflammatory bowel diseases, mild infections, parasitic infections and stressful life events. Post-inflammatory IBS (PI-IBS) normally occurs immediately after bacterial dysentery that is associated with *Campylobacter, Salmonella, Shigella*, protozoa, nematode or viral infections (El-Salhy, 2012). Even after the inflammation subsides, the infected intestinal segments exhibit altered motor functions and hypersensitivity. Most patients completely recover after acute infection, but effective pharmacotherapy for the exceptional ten percent of patients is lacking (Pasricha, 2007; Spiller and Garsed, 2009). Currently, the only therapeutic regimen involves relieving symptoms, such as abdominal cramping, bloating, diarrhea and constipation. Recently, the ideas that IBS is a dysfunctional disorder of the brain-gut axis or that IBS is due to abnormalities in the immune system have been widely accepted (Stark et al., 2007).

Because the pathogenesis of IBS is multifactorial, various types of stimuli have been employed to establish an IBS animal model. Chemical irritation of the colon is still a classical symptom-generating stimulus for establishing animal models of acute IBS. After acetic acid-induced inflammatory colitis subsides, the rats have visceral hypersensitivity and altered defecation patterns, which are the typical manifestations of IBS, in the absence of detectable disease (Dai et al., 2012). Accumulative evidence indicates that the gut microbiota are crucial for regulating the intestinal function and inflammation observed in IBS (Carroll et al., 2010). Intestinal bacterial overgrowth and gut dysbiosis are frequently observed in IBS patients (Posserud et al., 2007; Kim et al., 2012). Numerous studies have demonstrated decreased populations of *Lactobacillus* and *Bifidobacterium* and an increased presence of *Veillonella, Clostridium coccoides, Streptococci*, and *Coliforms* in IBS patients (Malinen et al., 2005; Kassinen et al., 2007; Kang and Lee, 2014). Thus, therapies aiming to normalize or regulate the unbalanced gut microbiota, such as dietary interventions (Chey, 2016), probiotics (Han et al., 2016) and antibiotics (Acosta et al., 2016), may have therapeutic potential for treatment of IBS.

Wuji Wan, comprising *Rhizoma Coptidis, Fructus Evodiae Rutaecarpae*, and *Radix Paeoniae Alba*, is a classical formula of Traditional Chinese Medicine (TCM) that has been employed in treating gastrointestinal disorders for hundreds of years. Currently, Wuji Wan is an OTC drug and has been recorded in the Chinese Pharmacopeia. Our previous studied showed that Wuji Wan could effectively ameliorate abdominal pain, colonic motility (CM) abnormality and visceral hypersensitivity in PI-IBS rats (Wang et al., 2013). However, evidence for how Wuji Wan mediates its effects on the gut microbiota is lacking for IBS treatment. Thus, the aim of this study was to elaborate on the alterations to the gut microbiota in the PI-IBS rat after Wuji Wan treatment and explore the possible mechanism.

## Results

### Effects of Wuji Wan on Colonic Motility

**FIGURE 1:**
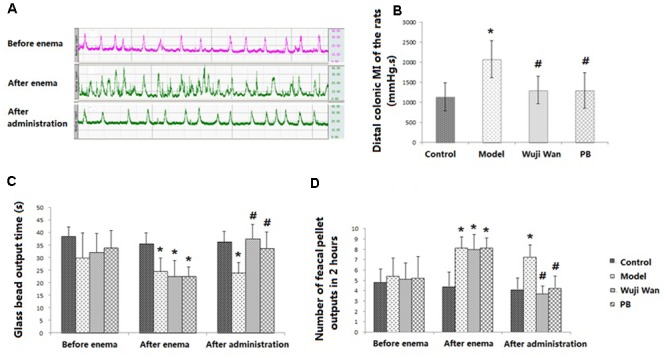
Evaluations of CM. **(A)** The representative curve of colonic movement in the Wuji Wan group. **(B)** The distal colonic MI of the rats (mean ± SD, *N* = 7). **(C)** Time of glass bead output (s) (mean ± SD, *N* = 7); **(D)** Number of fecal pellet outputs in 2 h (mean ± SD, *N* = 7). ^∗^*P* < 0.05 compared with the control group. ^#^*P* < 0.05 compared with the model group.

Representative curves of colonic movement before enema, after enema and after administration of Wuji Wan in the Wuji Wan group are shown in **Figure 1A**. These rhythmic waves of the distal colon exhibited an acceleration, became disordered after the enema intervention and recovered to normal with Wuji Wan treatment. The results of the distal colonic motility index (MI) (**Figure 1B**) also confirmed the effect of Wuji Wan and of the positive control, pinaverium bromide (PB), on PI-IBS, whereby a reduction in the colonic MI to normal was observed. As shown in **Figures 1C,D**, after the enema, the time of the glass bead output was significantly shortened, and the number of fecal pellet outputs over 2 h was significantly increased.

### Impact of Wuji Wan on Weight, Water Content and Mucin in Fecal Pellets

**Table 1** shows the wet weights, water contents and Mucin 2 (MUC2) levels in the fecal pellets from each group of rats. No significant differences were observed between the weights of the fecal pellets between the groups. The model process caused the water content of the fecal pellets to significantly increase (*P* < 0.001) and the concentration of MUC2 to significantly decrease (*P* < 0.05). After administration of Wuji Wan and PB, these changes were reversed to baseline.

**Table 1:** Effects of Wuji Wan and PB on the fecal pellet wet weight and water content in rats.

Group	Fecal pellet wet weight (mg)	Water content (%)	MUC2 (ng/g)
Control	300.25 ± 16.59	41.21 ± 3.66	3.15 ± 0.84
Model	336.25 ± 34.59	59.08 ± 3.03^∗∗^	1.52 ± 0.33^∗∗^
Wuji Wan	283.21 ± 45.89	40.31 ± 5.15^#^	2.51 ± 0.79^#^
PB	300.56 ± 28.56	42.96 ± 4.98^#^	2.37 ± 0.77^#^

*The results are expressed as the mean ± SEM of 7 animals per group. Statistical comparisons were performed using one-way ANOVA with the SPSS 19.0 software. ^∗∗^*P* < 0.01 vs. control group. ^#^*P* < 0.05 vs. model group.*

### Overall Structural Modulation of the Gut Microbiota after Wuji Wan Treatment

First, we used a bar-coded Illumina sequencing platform to analyze the structural changes in the gut microbiota for the control, model and Wuji Wan treatment groups. In total, 647 844 raw and 480 874 high-quality sequences were obtained from 21 samples with an average of 22 899 ± 5 326 reads per sample. The most abundant phyla included *Firmicutes* and *Bacteroidetes*. As revealed by taxon-based analysis, the abundances of the phyla *Bacteroidetes* (*P* < 0.05, FDR = 0.020), *Proteobacteria* (*P* < 0.05, FDR = 0.005) and *Verrucomicrobia* (*P* < 0.05, FDR = 0.007) were significantly higher in the Wuji Wan group than in the model group (Supplementary Table S1). The rarefaction and Shannon diversity curves revealed that most of the diversity had already been captured, although new rare phylotypes could be expected with additional sequencing (Supplementary Figure S1). Wuji Wan was associated with a significant decrease in the bacterial diversity of the gut microbiota in the rarefaction curve (*P* < 0.001 and *P* < 0.001 compared to the control and model groups) and Shannon analysis (*P* < 0.05 and *P* < 0.05 compared to the control and model groups) (Supplementary Figure S1).

**FIGURE 2:**
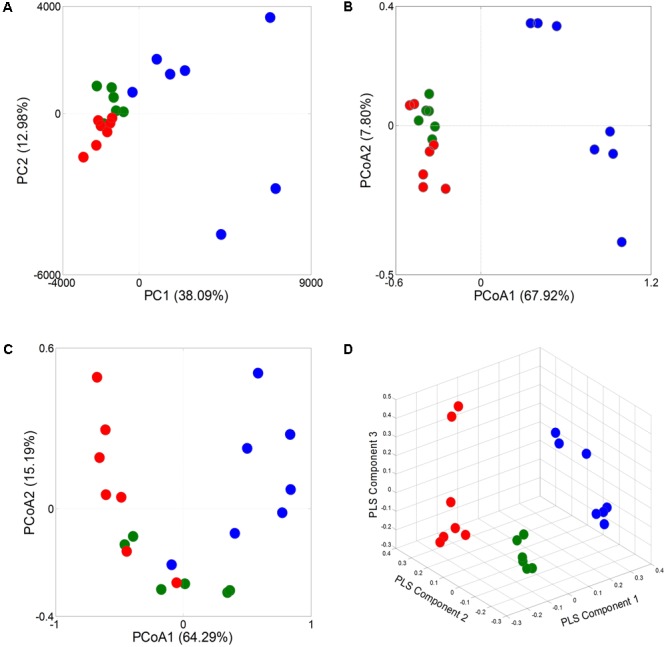
Responses of overall structure of the gut microbiota to Wuji Wan in rats. **(A)** PCA score plot. **(B)** PCoA score plot based on unweighted UniFrac metrics. **(C)** PCoA score plot based on weighted UniFrac metrics. **(D)** PLS-DA score plot.

A principal component analysis (PCA) revealed that the gut microbiota structure changed significantly in response to Wuji Wan administration. Wuji Wan-related differences were mainly observed along the first principal component (PC1), which accounted for the largest proportion (38.09%) of total variation (**Figure 2A**). Similar patterns were observed in the unweighted/weighted UniFrac principal coordinates analysis (PCoA) (**Figures 2B,C**), and host-gut microbe networks (Supplementary Figure S2), especially in the partial least squares-discriminate analysis (PLS-DA; **Figure 2D**).

### Key Phylotypes Responding to Wuji Wan Treatment

**FIGURE 3:**
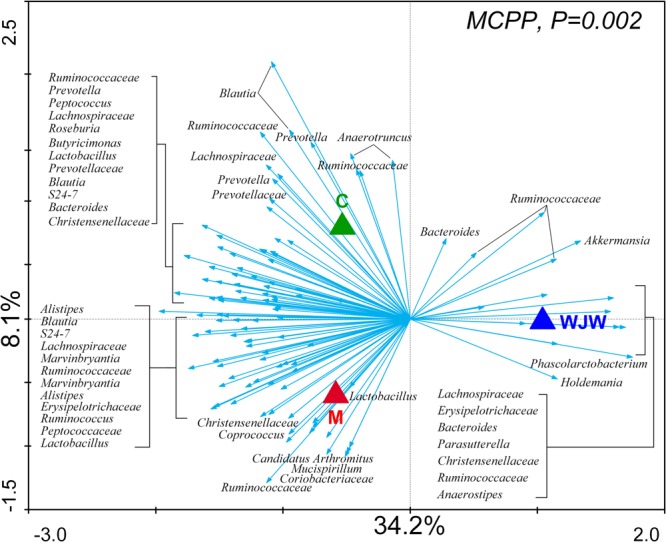
Distance triplot of the RDA of the gut microbiota. Nominal environmental variables [C (blank control), M (model control), and WJW (Wuji Wan treatment)] are indicated by triangles. A total of 116 OTUs that had at least 42% of the variability in their values explained by the first two axes are indicated by arrows. The relative best-fit species are labeled with taxonomic names (genus or family names). Upper left, *P*-value obtained by MCPP.

To identify key phylotypes of the gut microbiota that responded to the modeling process and Wuji Wan treatment, a redundancy analysis (RDA) was used to analyze the sequencing data. The major differences in the gut microbiota structure corresponded to the Wuji Wan treatment along the first ordination axis, which explained 34.2% of the total variability (**Figure 3**). Both the IBS model process and Wuji Wan administration led to significant changes in the gut microbiota structure, as validated by the Monte Carlo permutation procedure (MCPP) (*P* = 0.002).

**FIGURE 4:**
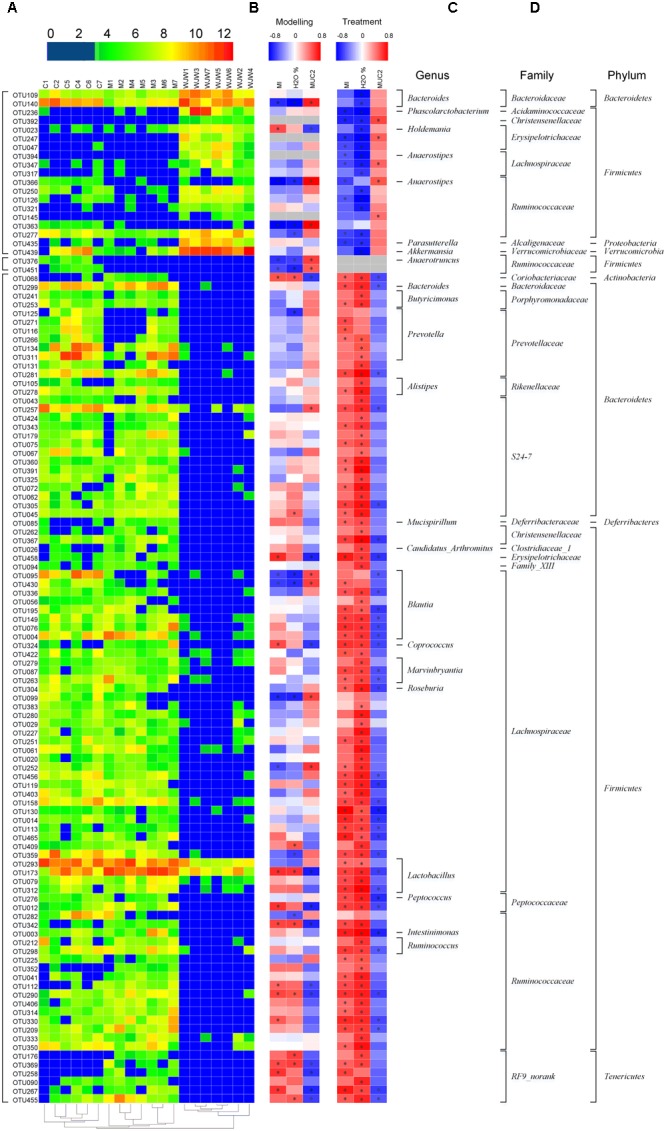
A total of 116 OTUs whose abundances were changed during the model construction process and Wuji Wan treatment according to the RDA. **(A)** Heatmap of the abundances of 116 OTUs. Rows correspond to 19 enriched OTUs and 97 reduced OTUs in the Wuji Wan group compared to those in the blank control. **(B,C)** Correlation between the changed OTUs and host parameters, including CM (MI), the water content and the MUC2 concentration of the fecal pellet, during the modeling and treatment process, respectively. The rows correspond to the OTUs with the IDs shown on the left, and the columns correspond to the parameters related to CM activity, feces characteristics and mucin 2 (MUC2) secretion from goblet cells. Red and blue indicate a positive and negative association, respectively. The intensity of the colors represents the degree of association between the OTU abundances and host parameters as assessed with the Spearman’s correlations. The black dots in the blue/red cells indicate that the associations were significant (*P*-value < 0.05). **(D)** The taxonomies of the OTUs (genus, family and phylum) are depicted on the right.

A total of 116 operational taxonomic units (OTUs) were identified that were associated with the model construction process and Wuji Wan treatment according to the RDA (**Figure 4**). For the treatment, 19 OTUs that were enriched by Wuji Wan included OTUs belonging to *Bacteroides* (*n* = 2), *Phascolarctobacterium* (n = 1), *Holdemania* (*n* = 1), *Anaerotruncus* (n = 1), *Anaerostipes* (n = 1), *Parasutterella* (n = 1), *Akkermansia* (n = 1), unclassified *Erysipelotrichaceae* (n = 2), unclassified *Lachnospiraceae* (*n* = 2), and unclassified *Ruminococcaceae* (*n* = 6). Most of these enriched OTUs showed significant negative correlations with the MI and water content of the feces, and 5 OTUs correlated with MUC2 positively. Another 97 OTUs were eliminated or decreased by Wuji Wan; several of these OTUs belonged to *Lactobacillus* (*n* = 4) and *Prevotella* (*n* = 6), which were markedly decreased.

Additionally, a taxon-based analysis at the genus level showed that the relative abundances of *Akkermansia* (*P* = 0.002, FDR = 0.025) and *Bacteroides* (*P* < 0.001, FDR = 0.006) were significantly higher after Wuji Wan treatment, whereas the abundances of *Lactobacillus* (*P* < 0.001, FDR = 0.003) and *Prevotella* (*P* = 0.033, FDR = 0.158) were significantly decreased (**Table 2** and Supplementary Figure S3). Notably, the abundance of *Akkermansia* was increased 11.2- and 234.1-fold more than the control and model groups after Wuji Wan treatment. The taxon-based results were also confirmed by the LEfSe analysis (Supplementary Figure S4).

**Table 2:** Significantly different genera between groups as revealed by taxon-based comparisons.

Genus	Blank control	Model control	WJW treatment	*P*	FDR
*Adlercreutzia*	0 ± 0.01	0.02 ± 0.02	0 ± 0	0.041	0.168
*Akkermansia*	1.67 ± 2.43^a^	0.08 ± 0.12^a^	18.73 ± 15.17^b^	0.002	0.025
*Allobaculum*	0 ± 0.01^ab^	0.02 ± 0.02^a^	0 ± 0^b^	0.026	0.135
*Bacteroides*	10.67 ± 4.75^a^	4.37 ± 2.86^a^	27.73 ± 12.78^b^	0.000	0.006
*Butyricimonas*	0.2 ± 0.23^a^	0.12 ± 0.09^ab^	0 ± 0^b^	0.045	0.168
*Christensenella*	0.01 ± 0.01^a^	0.03 ± 0.05^a^	0.11 ± 0.07^b^	0.002	0.025
*Desulfovibrio*	0.07 ± 0.05^a^	0.08 ± 0.1^a^	0.28 ± 0.15^b^	0.004	0.029
*Enhydrobacter*	0 ± 0^a^	0.01 ± 0.01^b^	0 ± 0^a^	0.003	0.026
*Erysipelotrichaceae_Incertae_Sedis*	0.12 ± 0.11^a^	0.23 ± 0.27^a^	0.88 ± 0.68^b^	0.011	0.067
*Holdemania*	0.01 ± 0.01^a^	0.02 ± 0.04^a^	0.46 ± 0.32^b^	0.000	0.011
*Lactobacillus*	22.19 ± 7.48^a^	35.31 ± 8.04^b^	8.14 ± 8.59^c^	0.000	0.003
*Mucispirillum*	0 ± 0.01^a^	0.03 ± 0.03^b^	0 ± 0^a^	0.006	0.041
*Parasutterella*	0.06 ± 0.04^a^	0.02 ± 0.01^a^	1.49 ± 1.12^b^	0.001	0.018
*Peptococcus*	0.05 ± 0.04^a^	0.03 ± 0.03^ab^	0 ± 0^b^	0.019	0.105
*Prevotella*	10.59 ± 10.96^a^	4.96 ± 4.7^ab^	0.01 ± 0.01^b^	0.033	0.158
*Pseudomonas*	0 ± 0^a^	0.06 ± 0.06^b^	0.02 ± 0.02^ab^	0.043	0.168
*Ruminococcus*	1.7 ± 1.36	2 ± 2.1	0.03 ± 0.08	0.047	0.168

*The results are expressed as the mean ± SEM of 7 animals per group. Statistical comparisons were performed using one-way ANOVA and Tukey’s *post hoc* range test for multiple comparisons at a significance level of *P* < 0.05.*

### Influence of Wuji Wan Administration on Goblet Cells in the Colon

**FIGURE 5:**
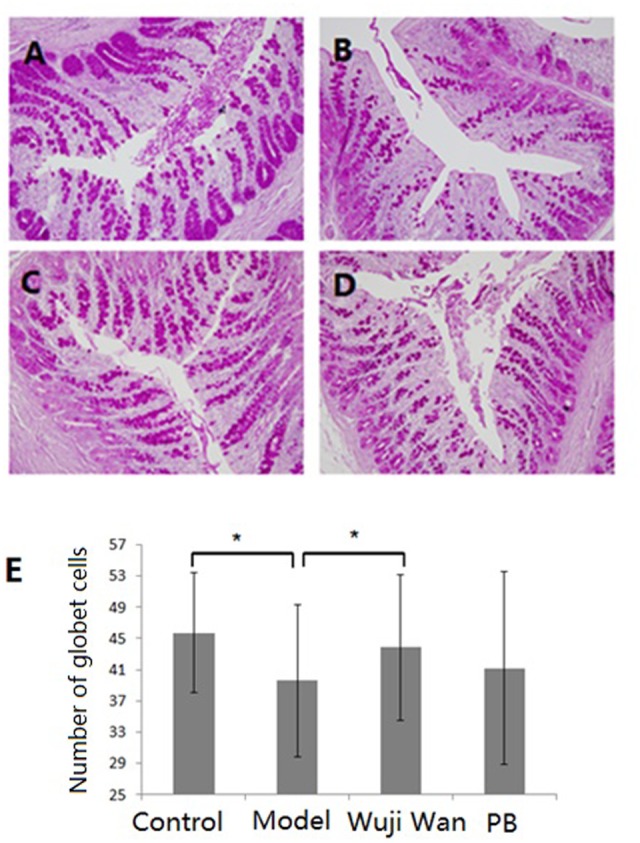
Influence of Wuji Wan administration on goblet cells in the colon. Photomicrographs of distal colons from the control group **(A)**, model group **(B)**, Wuji Wan group **(C)** and PB group **(D)** by H&E staining. TEM × 400. **(E)** The goblet cell count of the colonic tissue. ^∗^*P* < 0.05 compared with the control group. ^#^*P* < 0.05 compared with the model group.

Haematoxylin and eosin (H&E) staining of the lamina propria and submucosa was observed by microscopy. The intestinal epithelium structure and glandular arrangement were clear and showed integrity. No remarkable inflammatory features were observed in the colons of the normal or IBS rats by 40× light microscopy. **Figure 5** shows the distribution of the goblet cells between the columnar epithelial cells of the duodenum in the colon. Goblet cells are unicellular glands that secrete mucus, which acts as a lubricant in the colonic lumen. Goblet cells stain easily with the Periodic Acid-Schiff (PAS) stain, which colors the cells magenta during preparation of the microscopy samples. As shown in **Figure 5E**, the quantity of the goblet cells in the IBS model group was remarkably decreased, and Wuji Wan reversed this change.

### Effects of Wuji Wan on Tight Junctions

**FIGURE 6:**
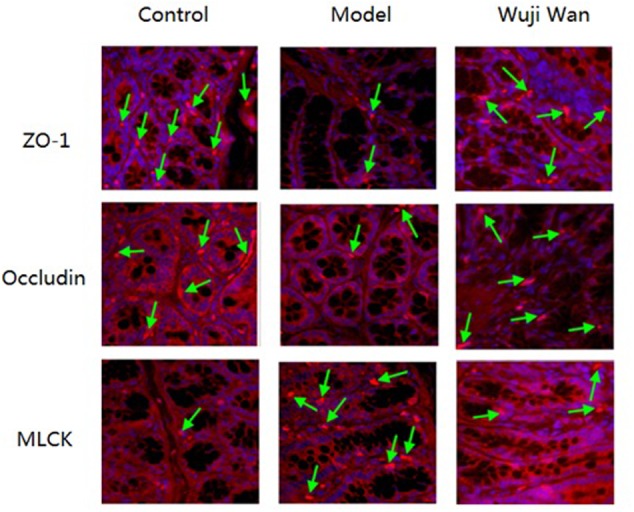
Effects of Wuji Wan on tight junctions. Expression of tight junction proteins (Occludin and ZO-1) and MLCK in the rat colon.

Immunofluorescence localization by confocal microscopy showed that ZO-1, Occludin and MLCK localized to the edges of the intestinal epithelial cells with honeycomb or linear shapes (**Figure 6**). Compared with the control group, the PI-IBS rats showed significantly decreased abundance of ZO-1 and Occludin and markedly increased abundance of MLCK. However, after treatment with Wuji Wan, the downregulated abundance of ZO-1 and Occludin increased significantly. The abundance of MLCK decreased to the normal level.

## Discussion

Irritable bowel syndrome is a prevalent functional gastrointestinal disorder that is characterized by abdominal pain and a change in the consistency of the stool. Increasing evidence has suggested that inflammation plays a role in the pathogenesis of IBS. In this study, after the inflammation subsided, the IBS rats suffered colitis CM functional disorder and diarrhea, making this animal model appropriate for studying IBS.

Although the etiology of IBS remains unclear, accumulative evidence has recently demonstrated the pivotal role of the gut microbiota in the pathogenesis of IBS. Biodiversity and temporal stability are diminished in the fecal and mucosal microbiota of IBS patients (Codling et al., 2010; Rinttilä et al., 2010; Jalankatuovinen et al., 2014). The phyla of *Bacteroidetes* (Rajilić–Stojanović et al., 2011) and *Verrucomicrobia* (Rigsbee et al., 2012) and the genera of *Bifidobacterium* (Kerckhoffs et al., 2009) are markedly decreased in IBS patients. Our results in IBS rats are in good agreement with those showed above in clinical. The level of *Bacteroidetes* decreased from 33.98 ± 15.78% to 18.43 ± 10.08%, and *Verrucomicrobia* decreased from 1.43 ± 2.3% to 0.08 ± 0.12% in the IBS rats in our current study. In addition, we also found that several genera, such as *Adlercreutzia, Allobaculum, Corynebacterium, Lactobacillus*, and *Parasporobacterium*, were in full bloom in the IBS rats. This disturbed intestinal ecology spurred the emergence of symptoms in the IBS rats.

A previous strategy for regulating human intestinal flora involved taking probiotic strains, such as *Bifidobacterium* and *Lactobacillus*, as a dietary supplement to adjust the abundances and biological structure of the host intestinal microflora. Evidence suggests that probiotics contribute to the relief of overall symptoms in IBS patients, especially for diarrhea-predominant IBS, reduction in bloating/distension and improvement of bowel movement frequency/consistency (Hungin et al., 2013). However, utilization of probiotic therapy is restricted due to a lack of knowledge regarding the effective dosage and the limited action times of probiotic strains. Because the composition of the microbiota is regulated by ingesta, prebiotics (dietary substances such as indigestible fructo-oligosaccharides and galacto-oligosaccharides) and synbiotics (products containing a synergistic combination of prebiotics and probiotics) are used to selectively promote the growth of beneficial bacteria in the gut. Due to the indigestible characteristic of prebiotics, their benefit is dose-dependent. High levels of prebiotics were revealed to counteract problems such as bloating and flatulence (Olesen and Gudmand-Høyer, 2000). Until now, no randomized controlled trials have been conducted to explore the therapeutic effect of TCM on IBS. In the present study, Wuji Wan exerted a significant effect on regulating intestinal bacteria. Wuji Wan reversed dysbiosis in the microbiota communities of the IBS rats. The TCM formula comprises several herbal ingredients.

The influence of a formula on the gut microbiota is the result of the comprehensive balance of each herb in the prescription. The structure of gut microbiota is modulated by different ingesta, among which TCM is the equivalent of fertilizer to affect some favorable specie in gut lumen. As shown in our study, the influences of Wuji Wan formula on intestinal flora were different from *Rhizoma Coptidis* (Xu et al., 2015) and berberine (Zhang et al., 2015), the major herb and most abundant chemical component in this formula. Our study suggests that gut microbiota might be involved in the effect of TCM formula. TCM formulas have the potential to redress the dysbiosis associated with the disease.

The more abundant phyla, namely, *Firmicutes, Bacteroidetes* and *Actinobacteria*, have presences and diversities that vary between high and low in the gut microbiota of patients with IBS (Bennet et al., 2015). The majority of IBS patients have an altered microbiota composition with increased *Firmicutes* and decreased *Bacteroidetes*, whereas the remaining patients had a normal-type gut microbiota composition (Jeffery et al., 2012). Moreover, *Bacteroidetes* is the second most abundant phylum in the gut and is over-represented in PI-IBS patients (Jalankatuovinen et al., 2014). However, in our results, the level of *Bacteroides* decreased from 10.67 ± 4.75% to 4.37 ± 2.86%. Previous studies have suggested a correlation between IBS and *Lactobacillus*. However, their findings are inconsistent (Bennet et al., 2015). In this study, the perturbed *Lactobacillus* community in the IBS rats was restored by Wuji Wan administration.

The discrepancies in the microbiota suggest that microbial dysbiosis is crucial in the pathogenesis of IBS. However, as described above, the alterations in the intestinal flora based on phylum or class classification were various, contradictory and confusing. An increasing number of researchers are realizing that disease-relevant bacterial functions may well be strain-specific (Zhang and Zhao, 2016) and that a dissection of the contribution of the gut microbiota to specific diseases should be performed to determine the exact species or strains.

*Akkermansia* was the markedly altered genus after Wuji Wan treatment. *Akkermansia muciniphila* is the single and type species of the genus *Akkermansia*, which first proposed in 2004 as a mucin-degrading, anaerobic Gram-negative bacterium that resides in the mucus layer (Derrien et al., 2004). Several studies have suggested a potential negative correlation between *A. muciniphila* and IBS (Rajilić–Stojanović et al., 2011; Saulnier et al., 2011). However, no direct evidence has been provided. Some exogenous factors may influence the abundance of *A. muciniphila*. These factors include diet, food supplements, TCM and drugs. The abundance of *A. muciniphila* was increased by 100-fold in genetically obese mice after administration of prebiotics (oligofructose) (Everard et al., 2011). Recent studies have demonstrated that the proportion of *A. muciniphila* increased and was accompanied by weight loss after feeding metformin, green tea, grapes and grape polyphenols to obese mice (Axling et al., 2012; Baldwin et al., 2015; Roopchand et al., 2015; Zhou et al., 2016). Additionally, caffeic acid dramatically increased the proportion of the mucin-degrading bacterium *A. muciniphila* in dextran sulfate sodium-induced colitis mice (Zhang et al., 2016). In the present study, we observed that the abundance of *A. muciniphila* was 20-fold lower in the IBS rats than in the normal rats and that the abundance was 234-fold higher in the IBS rats that received Wuji Wan treatment. The layer of mucus lining in the intestinal tract serves as a lubricant and physiological barrier between the luminal contents and mucosal surface. The abundant *A. muciniphila* in IBS rats may play a key role in maintaining the integrity of the mucin layer. However, whether Wuji Wan treatment increases the abundance of *A. muciniphila* by providing the main source of energy for this bacterium and thereby favoring its growth is unknown. Alternatively, whether the increase in *A. muciniphila* increases mucus production and degradation remains to be determined. Mucin 2 (MUC2) is secreted from goblet cells in the epithelial lining into the lumen of the large intestine. In the mucin layer, MUC2, along with small amounts of mucin-related proteins and water, polymerizes into a gel that provides an insoluble mucous barrier that serves to protect the intestinal epithelium. The histological damage in the colons of MUC2 (+/-) mice is more severe than that in the colons of wild-type [MUC2(+/+)] mice, which shows that MUC2 deficiency leads to a vulnerable mucus barrier and inflammation of the colon (Van der Sluis et al., 2006). Our results showed that the level of MUC2 in rat faeces was reduced by nearly one-half in the IBS rats. After treatment for 7 days with Wuji Wan, this decrease was reversed.

The gel-forming MUC2 mucins are secreted by goblet cells, which are glandular cells in the intestinal mucosal epithelium. Histological findings indicate that the number of goblet cells in the colonic epithelia of IBS rats was significantly reduced and that less mucin was stored in goblet cells. However, whether the change in mucus is causative or secondary to proliferation of *A. muciniphila* remains unclear. Treatment with some supplements, such as pomegranate (Li et al., 2015), oligofructose (Kleessen et al., 2003; Kleessen and Blaut, 2005) or *A. muciniphila* (Everard et al., 2013), significantly increases the number of goblet cells and the thickness of the mucus layer, which may contribute to gut barrier function restoration. Based on the present research, a Wuji Wan treatment-associated increase of *A. muciniphila* in the colon may indeed confer some degree of protection from the negative consequences of IBS.

Tight junction proteins, including Occludin and ZOs, are crucial for sealing the spaces between individual epithelial cells and maintaining the integrity of the epithelium. Loss of Occludin leads to an increase in gut permeability, whereas a deficiency in ZO-1 can interrupt the assembly of tight junctions by inhibiting the recruitment of other components. Previous studies have demonstrated that the expression levels of Occludin and ZO-1 are increased in the ileum of the Apoe-/- mouse after *A. muciniphila* treatment. Treatment with inoculating medium of *A. muciniphila* directly increases the expression levels of these two proteins in intestinal epithelial cells (Li et al., 2016). Phosphorylation of myosin light chain (MLCK) induces cytoskeletal contraction, which is an essential factor for the destruction of the intestinal epithelial barrier. Inhibiting the expression of MLCK *in vitro* restores the barrier function (Fazal et al., 2005). However, the causal relationship between the increased proportion of *A. muciniphila* and amelioration of tight junctions in IBS remains to be further elucidated.

In summary, our findings have demonstrated that Wuji Wan treatment increases the abundance of *A. muciniphila*, restores the mucus barrier and mitigates tight junctions. However, the underlying mechanisms that account for the Wuji Wan treatment-induced *A. muciniphila* increase require further investigation. Nonetheless, regulating the gut microbiota using TCM may become a potential therapeutic strategy for IBS.

## Materials and Methods

### Chemicals and Reagents

Milli-Q water (Bedford, MA, United States) was used throughout the study. Dried roots (3000 g) of *Rhizoma Coptidis* were extracted twice with water and 50% ethanol. The solution was evaporated in vacuum to yield a brown residue (579 g). Dried seeds (600 g) of *Fructus Evodiae Rutaecarpae* were extracted twice with water and 85% ethanol. The solution was evaporated in vacuum to yield a brown residue (106 g). The dried root (3000 g) of *Radix Paeoniae Alba* was pulverized and extracted twice with boiling water. The aqueous solution was evaporated in vacuum to yield a pale-yellow powder (279 g). The Wuji Wan extract was a mixture of the above three extracts and was stored in a light-resistant container at 4°C before use. The qualitative chemical profile of this extract was analyzed by LC–MS as previously described (Gong et al., 2014; Chen et al., 2015). PB tablets were obtained from Abbott Healthcare SAS (Abbott, Chatillon Sur Chalaronne, France). ELISA kits for rat LPS and MUC2 were obtained from Invitrogen Co. (Invitrogen Co., Carlsbad, CA, United States). All other reagents were analytical grade and obtained from Beijing Chemical Reagent Co. (Beijing Chemical Reagent Co., Beijing, China).

### Animals

Female Sprague-Dawley rats (200 ± 20 g) that were supplied by Beijing Vital Laboratory Animal Technology (Beijing, China) were used. The rats were maintained under standard conditions of temperature, humidity, and light. A commercial rat chow (Beijing Keao Co., Beijing, China) and water were autoclaved before use. All studies were performed in accordance with the proposals of the Committee for Research and Ethical Issues of the International Association for the Study of Pain and were approved by the Animal Ethics Committee at the Institute of Chinese Materia Medica, China Academy of Chinese Medical Sciences (approval number: 20142001).

### Induction of PI-IBS in Rats

After an overnight fast, acute colonic inflammation was induced in 21 rats to generate a PI-IBS model by intracolonic instillation of 4% acetic acid (1 mL) with a silicone tube connected to an injector at 8 cm proximal to the anus for 30 s. Then, phosphate-buffered saline (1 mL) was instilled to dilute the acetic acid and rinse the colon. The control rats were handled identically to the other rats except that saline was instilled instead of 4% acetic acid. Seven days later, the front upper limbs, chest and front portion of the PI-IBS model rats were wrapped with adhesive tape for 1 h. The rats had free access to food and water, except when the procedure required deprivation.

### Grouping and Administration

**FIGURE 7:**
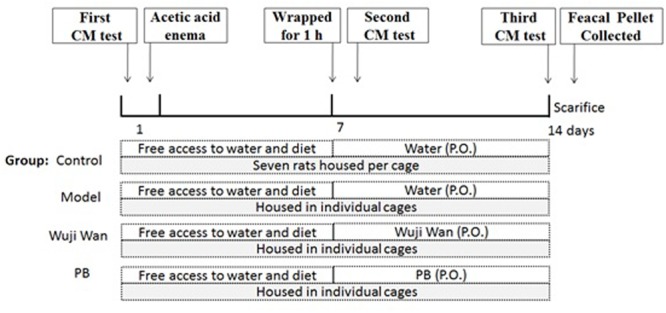
Schematic of the experimental design. The rats were divided into 4 groups: control, Model, Wuji Wan and PB. CM was assessed before beginning the induction of PI-IBS in rats. Wuji Wan or PB was administered by gavage during the second week. Following the third CM test, the rats were decapitated and sacrificed for assays of the gut microbiota and intestinal mucosal ultrastructure.

Twenty-eight rats were divided into 4 groups, with 7 rats per group. After the enema, rats were individually gavaged with sterile water (10 mL/kg), Wuji Wan extract (630 mg/kg, which was 4 times the clinical dosage) or PB (13.5 mg/kg) for 7 days. During the experiment, the rats in the control group were left undisturbed in their home cages in a separate room, whereas the other three groups of rats were housed in individual cages (one cage per rat). Body weights were measured daily. A schematic diagram of the experimental study design is shown in**Figure 7**.

### Evaluation of Colonic Motility (CM)

The rats were first placed into a cage with clean filter pads and given free access to the standard rat diet and water. Then, the number of feces defecated in 2 h were counted. The rats were then placed in a rubber capsule that contained water, which was connected to a multipurpose polygraph recording system (BIOPAC Co, United States) at 8 cm proximal to the anus. Colonic smooth muscle motility was recorded for 30 min. Quantification of CM was studied by calculating the MI. The MI was equivalent to the area under the curve of the motility recording and was calculated with a computer-assisted system (BIOPAC Co, United States). After the CM test, glass beads (diameter 3 mm) were placed into the rectum 3 mm deep from the anus. The time of glass bead output was measured.

### Sampling

At the end of week 2, all animals from the four groups were sacrificed, and their colons were removed for histological and lipid content analysis. Fecal samples were collected from the rectums of each rat after sacrifice, frozen immediately after sampling and stored at -80°C until the DNA extraction.

### Gut Microbiota Profiling

Fecal microbiota DNA was extracted using the E.Z.N.A.^®^ stool DNA Kit (Omega Bio-Tek, Norcross, GA, United States) according to the manufacturer’s protocols. The V3-V4 region of the bacteria 16S ribosomal RNA gene was amplified by PCR (95°C for 2 min, followed by 25 cycles at 95°C for 30 s, 55°C for 30 s, and 72°C for 30 s and a final extension at 72°C for 5 min) using primers 338F5′-barcode-ACTCCTACGGGAGGCAGCA)-3′ and 806R5′-GGACTACHVGGGTWTCTAAT-3′ (Dennis et al., 2013), where the barcode was an eight-base sequence unique to each sample. PCR reactions were performed in triplicate 20-μl mixtures containing 4 μl of 5× Fast Pfu buffer, 2 μl of 2.5 mmol/L dNTPs, 0.8 μl of each primer (5 μmol/L), 0.4 μl of Fast Pfu polymerase, and 10 ng of the template DNA.

Amplicons were extracted from 2% agarose gels and purified using the AxyPrep DNA Gel Extraction Kit (Axygen Biosciences, Union City, CA, United States) according to the manufacturer’s instructions and quantified using Quanti Fluor^TM^ -ST (Promega, United States). Purified amplicons were pooled into equimolar fractions and paired-end sequenced (PE300) on an Illumina MiSeq platform according to the standard protocols (Majorbio Bio-Pharm Technology Co. Ltd., Shanghai, China). The raw reads were deposited into the NCBI Sequence Read Archive database (Accession Number: SRP118771).

Raw FastQ files were demultiplexed and quality-filtered using QIIME (version 1.17) with the following criteria: (i) the 300-bp reads were truncated at any site receiving an average quality score < 20 over a 10-bp sliding window, discarding the truncated reads that were shorter than 50 bp; (ii) reads with exact barcode matching, a primer match with 2 nucleotide mismatches, and reads containing ambiguous characters were removed; and (iii) only sequences with overlaps longer than 10 bp were assembled according to their overlapping sequences. Reads that could not be assembled were discarded.

Operational taxonomic units were clustered with a 97% similarity cutoff using UPARSE (version 7.1)^1^, and chimeric sequences were identified and removed using UCHIME. The phylogenetic affiliation of each 16S rRNA gene sequence was analyzed with RDP Classifier^2^ against the SILVA (SSU115) 16S rRNA database using a confidence threshold of 70%.

### Fecal Pellet Water Content and MUC2 Concentration

Fecal pellets were then weighed (wet weight in mg), desiccated in an oven (50°C, 6 h), and weighed again (dry weight in mg). The fecal water content was calculated according to the following equation: Water content (%) = 100 (wet weight – dry weight)/wet weight. The ratio of the wet to dry weights was calculated and used as a marker of fecal water content. The concentration of MUC2 in the feces was examined with highly sensitive ELISA techniques.

### Histological Assessment

To examine the goblet cells and extent of colonic inflammation, the distal 10 cm of the descending colon was removed, placed in 10% formalin, and sent for histological processing. A cross-section of the colon wall was fixed in formalin, dehydrated in a graded alcohol series and xylene, embedded in paraffin, and cut serially into 4-mm sections to be stained with haematoxylin-eosin (H&E) and PAS. The coded slides were analyzed by Shuangrong Gao, a pathologist in the GLP laboratory of China Academy of Chinese Medical Sciences who was blinded to the source of the sample. The number of goblet cells was counted in 10 high-power fields (×400) of each coded slide, and the mean number of goblet cells was calculated.

### Immunofluorescence Analysis

Following antigen repair in 0.01 M citrate buffer, the tissues sections were blocked in goat serum. The tissue slices were incubated for 1 h with primary antibodies (mouse monoclonal anti-Occludin, rabbit polyclonal anti-ZO-1 or mouse monoclonal anti-MLCK antibodies) followed by incubation for 1 h with secondary antibodies (goat anti-rabbit IgG-PE). The slices were stained with DAPI. Fluorescence was examined under an Olympus FV-1000 laser scanning confocal microscope.

### Statistical Analyses

Data were expressed as the mean ± SD. Statistical comparisons were performed using one-way analysis of variance (ANOVA) with the SPSS 19.0 software. A *P*-value < 0.05 indicated statistical significance.

The overview of the gut microbiota was characterized with a PCoA. Multivariate analysis of variance was carried out to cluster the gut microbiota based on the Mahalanobis distance. A partial least squares discriminant analysis (PLS-DA) was used to establish the classification model based on the OTUs data. The correct prediction rate of the PLS-DA model was performed with leave-one-out cross-validation. Selection of significant OTUs that could distinguish different treatment groups was performed with the Martens’ uncertainty test and one-way ANOVA. Statistical significance was set at ^∗^*P* < 0.05 and ^∗∗^*P* < 0.01.

## Author Contributions

YC and XZ designed the experimental protocol. QY, YL, YD, YW, QL, XW, WC, and WQ carried out the animal surgeries and the sample collection. SG and ZG contributed to the histological examination and goblet cell counting. YC and SX carried out the data analysis and interpretation and wrote a draft of the paper. XZ and ZS contributed to the critical review of the paper. All authors have read, commented on, and approved of the manuscript.

## Conflict of Interest Statement

The authors declare that the research was conducted in the absence of any commercial or financial relationships that could be construed as a potential conflict of interest.

## Footnotes

1 http://drive5.com/uparse/

2 http://rdp.cme.msu.edu/

## References

[B1] Acosta A. Camilleri M. Shin A. Nord S. L. O’Neill J. Gray A. V. (2016). Effects of rifaximin on transit, permeability, fecal microbiome, and organic acid excretion in irritable bowel syndrome. *Clin. Transl. Gastroenterol.* 7 e173. 10.1038/ctg.2016.32 27228404 PMC4893683

[B2] Axling U. Olsson C. Jie X. Fernandez C. Larsson S. Ström K. (2012). Green tea powder and *Lactobacillus plantarum* affect gut microbiota, lipid metabolism and inflammation in high-fat fed C57BL/6J mice. *Nutr. Metab.* 9:105. 10.1186/1743-7075-9-105 23181558 PMC3538623

[B3] Baldwin J. Collins B. Wolf P. G. Martinez K. Shen W. Chuang C. C. (2015). Table grape consumption reduces adiposity and markers of hepatic lipogenesis and alters gut microbiota in butter fat-fed mice. *J. Nutr. Biochem.* 27 123–135. 10.1016/j.jnutbio.2015.08.027 26423887 PMC4933288

[B4] Bennet S. M. P. Öhman L. Simrén M. (2015). Gut microbiota as potential orchestrators of irritable bowel syndrome. *Gut Liver* 9 318–331. 10.5009/gnl14344 25918261 PMC4413965

[B5] Carroll I. M. Chang Y. H. Park J. Sartor R. B. Ringel Y. (2010). Luminal and mucosal-associated intestinal microbiota in patients with diarrhea-predominant irritable bowel syndrome. *Gut Pathog.* 2:19. 10.1186/1757-4749-2-19 21143915 PMC3018384

[B6] Chen Y. Li Y. Wang Y. Yang Q. Yu D. Weng X. (2015). Comparative pharmacokinetics of active alkaloids after oral administration of *Rhizoma coptidis* extract and Wuji Wan formulas in rat using a UPLC–MS/MS method. *Eur. J. Drug Metab. Pharmacokinet.* 40 67–74. 10.1007/s13318-014-0181-1 24577954

[B7] Chey W. D. (2016). Food: the main course to wellness and illness in patients with irritable bowel syndrome. *Am. J. Gastroenterol.* 111 366–371. 10.1038/ajg.2016.12 26856749

[B8] Codling C. O’Mahony L. Shanahan F. Quigley E. M. M. Marchesi J. R. (2010). A molecular analysis of fecal and mucosal bacterial communities in irritable bowel syndrome. *Dig. Dis. Sci.* 55 392–397. 10.1007/s10620-009-0934-x 19693670

[B9] Dai C. Guandalini S. Zhao D. H. Jiang M. (2012). Antinociceptive effect of VSL#3 on visceral hypersensitivity in a rat model of irritable bowel syndrome: a possible action through nitric oxide pathway and enhance barrier function. *Mol. Cell. Biochem.* 362 43–53. 10.1007/s11010-011-1126-5 22020749

[B10] Dennis K. L. Wang Y. Blatner N. R. Wang S. Saadalla A. Trudeau E. (2013). Adenomatous polyps are driven by microbe-instigated focal inflammation and are controlled by IL-10-producing T cells. *Cancer Res.* 73 5905–5913. 10.1158/0008-5472.CAN-13-1511 23955389 PMC4322779

[B11] Derrien M. Vaughan E. E. Plugge C. M. Vos W. M. D. (2004). *Akkermansia muciniphila* gen. nov., sp. nov., a human intestinal mucin-degrading bacterium. *Int. J. Syst. Evol. Microbiol.* 54(Pt 5) 1469–1476. 10.1099/ijs.0.02873-0 15388697

[B12] El-Salhy M. (2012). Irritable bowel syndrome: diagnosis and pathogenesis. *World J. Gastroenterol.* 18 5151–5163. 10.3748/wjg.v18.i37.5151 23066308 PMC3468846

[B13] Everard A. Belzer C. Geurts L. Ouwerkerk J. P. Druart C. Bindels L. B. (2013). Cross-talk between *Akkermansia muciniphila* and intestinal epithelium controls diet-induced obesity. *Proc. Natl. Acad. Sci*. *U.S.A.* 22110 9066–9071. 10.1073/pnas.1219451110 23671105 PMC3670398

[B14] Everard A. Lazarevic V. Derrien M. Girard M. Muccioli G. Neyrinck A. (2011). Responses of gut microbiota and glucose and lipid metabolism to prebiotics in genetic obese and diet-induced leptin-resistant mice. *Diabetes* 60 2775–2786. 10.2337/db11-0227 21933985 PMC3198091

[B15] Fazal F. Gu L. Ihnatovych I. Han Y. Hu W. Antic N. (2005). Inhibiting myosin light chain kinase induces apoptosis in vitro and in vivo. *Mol. Cell. Biol.* 25 6259–6266. 10.1128/MCB.25.14.6259-6266.2005 15988034 PMC1168802

[B16] Gong Z. Chen Y. Zhang R. Wang Y. Guo Y. Yang Q. (2014). Pharmacokinetic comparison of berberine in rat plasma after oral administration of berberine hydrochloride in normal and post inflammation irritable bowel syndrome rats. *Int. J. Mol. Sci.* 15 456–467. 10.3390/ijms15010456 24451127 PMC3907819

[B17] Han K. Wang J. Seo J. G. Kim H. (2016). Efficacy of double-coated probiotics for irritable bowel syndrome: a randomized double-blind controlled trial. *J. Gastroenterol.* 52 432–443. 10.1007/s00535-016-1224-y 27178566

[B18] Hungin A. P. S. Mulligan C. Pot B. Whorwell P. Agréus L. Fracasso P. (2013). Systematic review: probiotics in the management of lower gastrointestinal symptoms in clinical practice – an evidence-based international guide. *Aliment. Pharmacol. Ther.* 38 864–886. 10.1111/apt.12460 23981066 PMC3925990

[B19] Jalankatuovinen J. Salojärvi J. Salonen A. Immonen O. Garsed K. Kelly F. M. (2014). Faecal microbiota composition and host-microbe cross-talk following gastroenteritis and in postinfectious irritable bowel syndrome. *Gut* 63 1737–1745. 10.1136/gutjnl-2013-305994 24310267

[B20] Jeffery I. B. O’Toole P. W. Öhman L. Claesson M. J. Deane J. Quigley E. M. M. (2012). An irritable bowel syndrome subtype defined by species-specific alterations in faecal microbiota. *Gut* 61 997–1006. 10.1136/gutjnl-2011-301501 22180058

[B21] Kang N. L. Lee O. Y. (2014). Intestinal microbiota in pathophysiology and management of irritable bowel syndrome. *World J. Gastroenterol.* 20 8886–8897. 10.3748/wjg.v20.i27.8886 25083061 PMC4112865

[B22] Kassinen A. Krogius-Kurikka L. Mäkivuokko H. Rinttilä T. Paulin L. Corander J. (2007). The fecal microbiota of irritable bowel syndrome patients differs significantly from that of healthy subjects. *Gastroenterology* 133 24–33. 10.1053/j.gastro.2007.04.005 17631127

[B23] Kerckhoffs A. P. Samsom M. van der Rest M. E. de Vogel J. Knol J. Ben-Amor K. (2009). Lower bifidobacteria counts in both duodenal mucosa-associated and fecal microbiota in irritable bowel syndrome patients. *World J. Gastroenterol.* 15 2887–2892. 10.3748/wjg.15.2887 19533811 PMC2699007

[B24] Kim G. Deepinder F. Morales W. Hwang L. Weitsman S. Chang C. (2012). *Methanobrevibacter smithii* is the predominant methanogen in patients with constipation-predominant IBS and methane on breath. *Dig. Dis. Sci.* 57 3213–3218. 10.1007/s10620-012-2197-1 22573345

[B25] Kleessen B. Blaut M. (2005). Modulation of gut mucosal biofilms. *Br. J. Nutr.* 93 S35–S40. 10.1079/BJN20041346 15877893

[B26] Kleessen B. Hartmann L. Blaut M. (2003). Fructans in the diet cause alterations of intestinal mucosal architecture, released mucins and mucosa-associated bifidobacteria in gnotobiotic rats. *Br. J. Nutr.* 89 597–606. 10.1079/BJN2002827 12720580

[B27] Li J. Lin S. Vanhoutte P. M. Woo C. W. Xu A. (2016). *Akkermansia muciniphila* protects against atherosclerosis by preventing metabolic endotoxemia-induced inflammation in *Apoe*-/- mice. *Circulation* 133 2434–2446. 10.1161/CIRCULATIONAHA.115.019645 27143680

[B28] Li Z. Henning S. Lee R. Lu Q. Summanen P. Thames G. (2015). Pomegranate extract induces ellagitannin metabolite formation and changes stool microbiota in healthy volunteers. *Food Funct.* 6 2487–2495. 10.1039/c5fo00669d 26189645

[B29] Malinen E. Rinttilä T. Kajander K. Mättö J. Kassinen A. Krogius L. (2005). Analysis of the fecal microbiota of irritable bowel syndrome patients and healthy controls with real-time PCR. *Am. J. Gastroenterol.* 100 373–382. 10.1111/j.1572-0241.2005.40312.x 15667495

[B30] Olesen M. Gudmand-Høyer E. (2000). Efficacy, safety, and tolerability of fructooligosaccharides in the treatment of irritable bowel syndrome. *Am. J. Clin. Nutr.* 72 1570–1575. 11101487 10.1093/ajcn/72.6.1570

[B31] Pasricha P. J. (2007). Desperately seeking serotonin a commentary on the withdrawal of tegaserod and the state of drug development for functional and motility disorders. *Gastroenterology* 132 2287–2290. 10.1053/j.gastro.2007.04.057 17570201

[B32] Posserud I. Stotzer P. O. Björnsson E. S. Abrahamsson H. Simrén M. (2007). Small intestinal bacterial overgrowth in patients with irritable bowel syndrome. *Gut* 56 802–808. 10.1136/gut.2006.108712 17148502 PMC1954873

[B33] Rajilić–Stojanović M. Biagi E. Heilig H. G. Kajander K. Kekkonen R. A. Tims S. (2011). Global and deep molecular analysis of microbiota signatures in fecal samples from patients with irritable bowel syndrome. *Gastroenterology* 141 1792–1801. 10.1053/j.gastro.2011.07.043 21820992

[B34] Rigsbee L. Agans R. Shankar V. Kenche H. Khamis H. J. Michail S. (2012). Quantitative profiling of gut microbiota of children with diarrhea-predominant irritable bowel syndrome. *Am. J. Gastroenterol.* 107 1740–1751. 10.1038/ajg.2012.287 22986438

[B35] Rinttilä T. Lyra A. Krogiuskurikka L. Palva A. (2010). Real-time PCR analysis of enteric pathogens from fecal samples of irritable bowel syndrome subjects. *Gut Pathog.* 3:6. 10.1186/1757-4749-3-6 21518462 PMC3111350

[B36] Rivkin A. (2003). Tegaserod maleate in the treatment of irritable bowel syndrome: a clinical review. *Clin. Ther.* 25 1952–1974. 10.1016/S0149-2918(03)80198-4 12946544

[B37] Roopchand D. E. Carmody R. N. Kuhn P. Moskal K. Rojassilva P. Turnbaugh P. J. (2015). Dietary polyphenols promote growth of the gut bacterium *Akkermansia muciniphila* and attenuate gigh-fat diet-induced metabolic syndrome. *Diabetes* 64 2847–2858. 10.2337/db14-1916 25845659 PMC4512228

[B38] Saulnier D. M. Riehle K. Mistretta T. A. (2011). Gastrointestinal microbiome signatures of pediatric patients with irritable bowel syndrome. *Gastroenterology* 141 1782–1791. 10.1053/j.gastro.2011.06.072 21741921 PMC3417828

[B39] Spiller R. Garsed K. (2009). Postinfectious irritable bowel syndrome. *Gastroenterology* 136 1979–1988. 10.1053/j.gastro.2009.02.074 19457422

[B40] Stark D. Hal S. V. Marriott D. Ellis J. Harkness J. (2007). Irritable bowel syndrome: a review on the role of intestinal protozoa and the importance of their detection and diagnosis. *Int. J. Parasitol.* 37 11–20. 10.1016/j.ijpara.2006.09.009 17070814

[B41] Van der Sluis M. De Koning B. A. De Bruijn A. C. Velcich A. Meijerink J. P. Van Goudoever J. B. (2006). Muc2-deficient mice spontaneously develop colitis, indicating that MUC2 is critical for colonic protection. *Gastroenterology* 131 117–129. 10.1053/j.gastro.2006.04.020 16831596

[B42] Wang Y. Zhou S. Wang Y. Gong Z. Yang Q. Kan X. (2013). Influence of Wuji Wan in different compatibilities on colonic motility and 5-hydroxytryptamine contents in rats with post-infectious irritable bowel syndrome. *World J. Gastroenterol.* 21 1226–1233. 10.11569/wcjd.v21.i13.1226

[B43] Xu J. Lian F. Zhao L. Zhao Y. Chen X. Zhang X. (2015). Structural modulation of gut microbiota during alleviation of type 2 diabetes with a Chinese herbal formula. *ISME J.* 9 552–562. 10.1038/ismej.2014.177 25279787 PMC4331591

[B44] Zhang C. Zhao L. (2016). Strain-level dissection of the contribution of the gut microbiome to human metabolic disease. *Genome Med.* 8:41. 10.1186/s13073-016-0304-1 27098841 PMC4839137

[B45] Zhang X. Zhao Y. Xu J. Xue Z. Zhang M. Pang X. (2015). Modulation of gut microbiota by berberine and metformin during the treatment of high-fat diet-induced obesity in rats. *Sci. Rep.* 5:14405. 10.1038/srep14405 26396057 PMC4585776

[B46] Zhang Z. Wu X. Cao S. Wang L. Wang D. Yang H. (2016). Caffeic acid ameliorates colitis in association with increased *Akkermansia* population in the gut microbiota of mice. *Oncotarget* 7 31790–31799. 10.18632/oncotarget.9306 27177331 PMC5077976

[B47] Zhou Z. Y. Ren L. W. Zhan P. Yang H. Y. Chai D. D. Yu Z. W. (2016). Metformin exerts glucose-lowering action in high-fat fed mice via attenuating endotoxemia and enhancing insulin signaling. *Acta Pharmacol. Sin.* 37 1063–1075. 10.1038/aps.2016.21 27180982 PMC4973377

