# Supplementary material for: Wuji Wan Formula Ameliorates Diarrhea and Disordered Colonic Motility in Post-inflammation Irritable Bowel Syndrome Rats by Modulating the Gut Microbiota
Source: Front Microbiol. 2017 Nov 23;8:2307. doi: 10.3389/fmicb.2017.02307 (PMC5703868; doi:10.3389/fmicb.2017.02307)
Supplement: Supplementary file 1 [file Presentation_1.pdf]

Supplementary information

Supplementary results

Figure S1

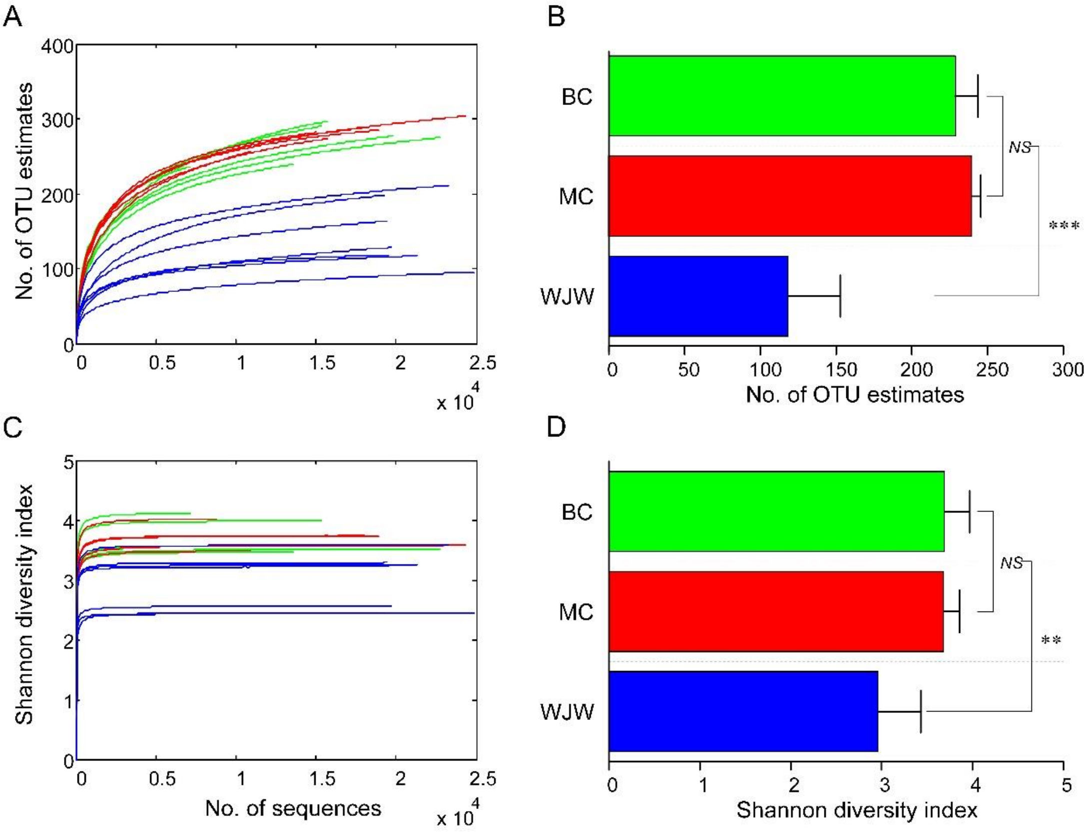

Figure S1 Rarefaction and Shannon analysis of each sequencing sample. BC, blank control; MC, model control, WJW, Wuji Wan treatment. (A) and (C) show the Rarefaction curves and Shannon index curves of each sample, respectively; (B) and (D) show the Rarefaction OTU estimates and Shannon index of each group. Calculations were performed after rarefying an equal number (7140 reads) of sequence reads for all samples. Values are expressed as means ± standard error.

Differences were assessed by one-way ANOVA/Turkey post hoc test and denoted as follows: * P<0.05; ** P<0.01; *** P<0.001; NS not significant.

Figure S2

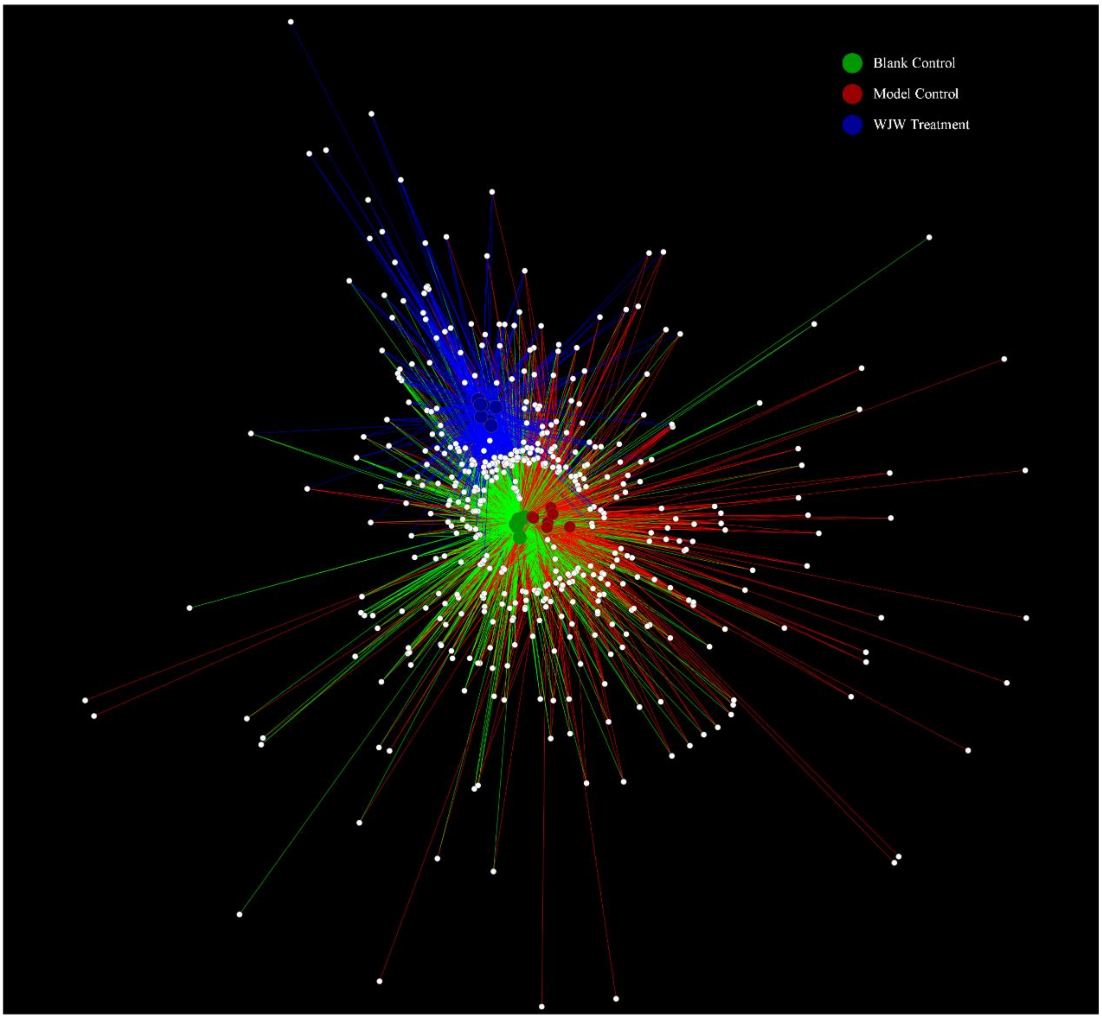

Figure S2 Simplified cartoon illustration of possible host-gut microbe networks.

Network diagrams are color-coded by type of treatment of the subjects.

Figure S3

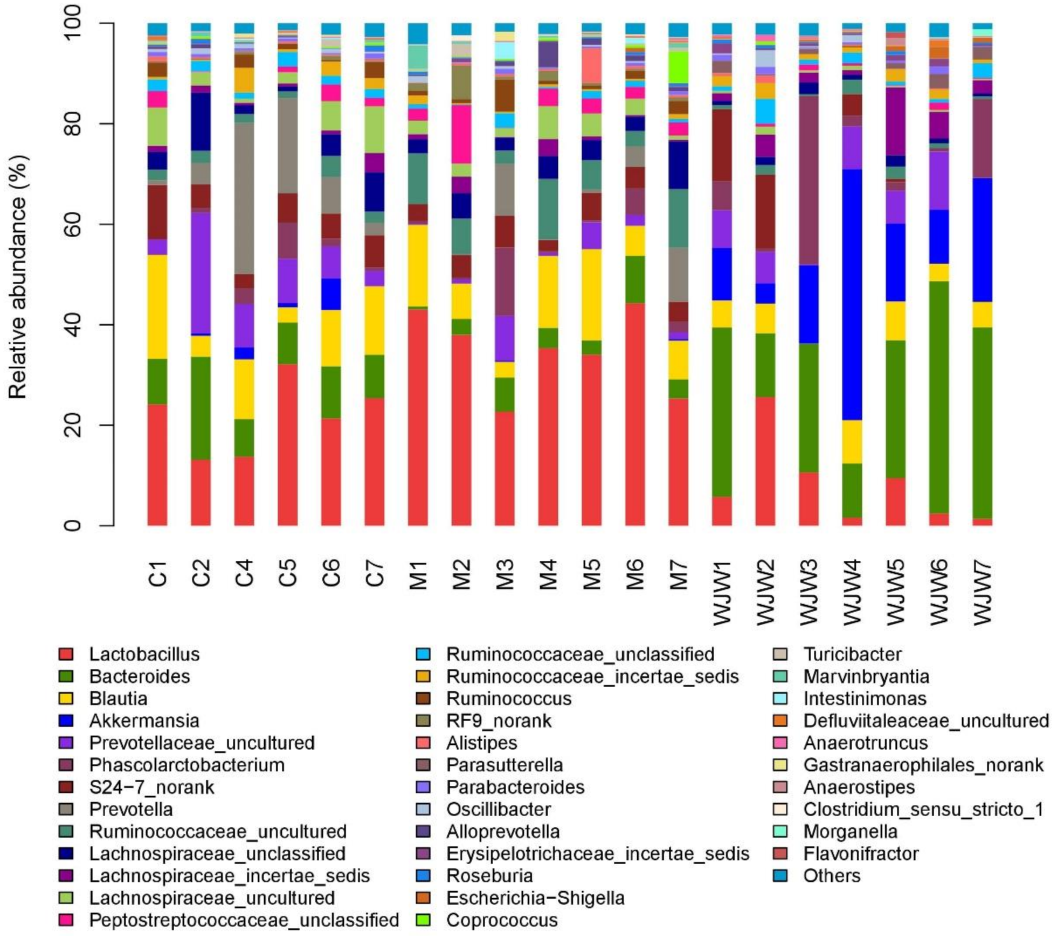

Figure S3 Summary of the bacterial taxa present in the gut community of each mouse. C1-7, blank control; M1-7, model control; WJW1-7, Wuji wan treatment. Each sample analyzed is indicated along the X-axis, the Y-axis indicates the relative abundance of each type (genus) of bacteria present in that gut community. A key to the bacteria taxa is listed at the bottom.

Figure S4

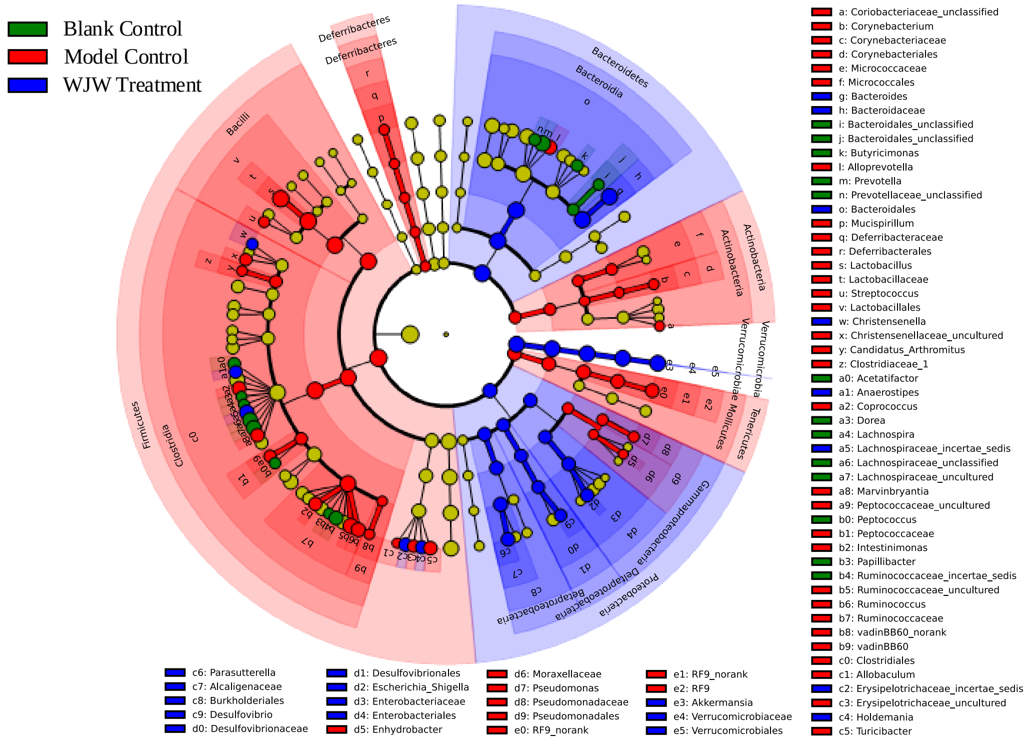

Figure S4 Habitat-specific taxa detected by LEfSe. Color indicates the rectum habitat in which each differential clade was most abundant.

Table S1

Table S1 Significantly different phyla between groups as revealed by taxon-based comparisons.

Phylum	Blank control	Model control	WJW treatment	P	FDR
Actinobacteria	0.05±0.06	0.27±0.22	0.16±0.15	0.078	0.111
Bacteroidetes	37.75±13.41[a]	18.43±10.08[b]	39.66±13.74[a]	0.010	0.020
Candidate_division_TM7	0±0	0.06±0.11	0±0	0.223	0.272
Cyanobacteria	0.23±0.31	0.31±0.69	0±0	0.430	0.430
Deferribacteres	0±0.01[a]	0.03±0.03[b]	0±0[a]	0.006	0.015
Elusimicrobia	0±0.01	0.01±0.01	0±0	0.245	0.272
Firmicutes	59.37±13.33[a]	78.49±9.32[b]	38.42±14.41[c]	0.000	0.000
Proteobacteria	0.54±0.3[a]	0.56±0.43[a]	3.02±1.88[b]	0.001	0.005
Tenericutes	0.37±0.3	1.75±2.24	0±0	0.062	0.103
Verrucomicrobia	1.67±2.43[a]	0.08±0.12[a]	18.73±15.17[b]	0.002	0.007

The results are expressed as the mean±SEM of 7 animals per group. Statistical comparisons were performed using one-way ANOVA and Tukey's post hoc range test for multiple comparisons at a significance level of $P<0.05$.
